# Clinical characteristics of *Plasmodium falciparum* infection among symptomatic patients presenting to a major urban military hospital in Cameroon

**DOI:** 10.1186/s12936-022-04315-2

**Published:** 2022-10-22

**Authors:** Daniel Z. Hodson, Yannick Mbarga Etoundi, Narcisse Mbatou Nghokeng, Raïhana Mohamadou Poulibe, Sonia Magne Djoko, Justin Goodwin, Glwadys Cheteug Nguesta, Tatiana Nganso, Jillian N. Armstrong, John J. Andrews, Elizabeth Zhang, Martina Wade, Carole Else Eboumbou Moukoko, Yap Boum, Sunil Parikh

**Affiliations:** 1 Yale School of Medicine, New Haven, USA; 2 Douala Military Hospital, Douala, Cameroon; 3 Douala Military Hospital School of Nursing, Douala, Cameroon; 4 Faculty of Medicine and Pharmaceutical Sciences, University of Douala, Douala, Cameroon; 5 Yale School of Public Health, New Haven, USA; 6 Department of Microbiology and Parasitology, University of Buea, Buea, Cameroon; 7 Malaria Research Service, Centre Pasteur of Cameroon, Yaoundé, Cameroon; 8 Epicentre, Yaoundé, Cameroon; 9 Faculty of Medicine and Biomedical Sciences, University of Yaoundé, Yaoundé, Cameroon

**Keywords:** Malaria, *Plasmodium falciparum*, Anaemia, Cameroon, Douala, Febrile illness, Urban

## Abstract

**Background:**

Urban malaria has received insufficient attention in the literature. The prevalence and clinical characteristics of *Plasmodium falciparum* infection amongst patients presenting with suspected malaria were investigated at a major urban hospital in Douala, Cameroon with a particular focus on anaemia.

**Methods:**

A cross-sectional, 18-week demographic and clinical survey was conducted of patients presenting to the Emergency Department of Douala Military Hospital with suspected malaria, largely defined by the presence or recent history of fever. Venous samples were tested for *P. falciparum* using rapid diagnostic tests and PCR, and anaemia was defined by haemoglobin level according to WHO definitions. Likelihood ratios (LR), odds ratios (OR), and population attributable risk percent (PARP) were calculated.

**Results:**

Participants were ages 8 months to 86 years, 51% were women (257/503), and all districts of Douala were represented. Overall, 38.0% (n = 189/497) were anaemic, including 5.2% (n = 26/497) with severe anaemia. Anaemia prevalence was significantly higher (OR: 2.20, 95% CI 1.41–3.45) among children < 15 years (53.1%, n = 52/98) compared to adults (34%, n = 133/392). *Plasmodium falciparum* was detected in 37.2% by nested PCR. Among all participants, several factors were associated with clinically significant LR for *P. falciparum* infection, including age 10–14 years (positive LR: 3.73), living in the island district of Douala VI (positive LR: 3.41), travel to any of three northern regions (positive LR: 5.11), and high fever > 40 °C at presentation (positive LR: 4.83). Among all participants, 8.7% of anaemia was associated with *P. falciparum* infection, while the PARP was 33.2% among those < 15 years of age and 81.0% among 10–14-year-olds.

**Conclusions:**

The prevalence of *P. falciparum* infection in the urban hospital was high. Mirroring trends in many rural African settings, older children had the highest positivity rate for *P. falciparum* infection. Anaemia was also common in all age groups, and for those 10–14 years of age, 80% of the risk for anaemia was associated with *P. falciparum* infection. Malaria rates in major urban population centres can be high, and more research into the multifactorial causes of anaemia across the age spectrum are needed.

**Supplementary Information:**

The online version contains supplementary material available at 10.1186/s12936-022-04315-2.

## Background

Malaria remains one of the most important global infectious diseases, with an estimated 241 million incident cases and 627,000 estimated malaria deaths in 2020 [1]. Malaria morbidity and mortality fall disproportionally on the African continent, with 95% of malaria cases and 96% of malaria deaths occurring in the World Health Organization’s (WHO) African Region in 2020 [1]. In sub-Saharan Africa, the risk of malaria has been traditionally considered to be significantly higher in rural than urban settings [2]. This increased risk has been attributed to multiple factors, including a lack of vectors ideally suited to urban environments, better housing, and improved access to care. However, many epidemiologic and demographic shifts have been occurring on the continent, including reports of shifting age burdens for malaria, emergence of unique risk factors for urban malaria, and recent reports of expanding ranges for the urban dwelling *Anopheles stephensi* vector [3–7].

Located in the WHO Central Africa region, Cameroon is one of the WHO’s designated high burden to high impact malaria countries with > 6.9 million malaria cases and 14,841 malaria deaths estimated for 2020 [1, 8, 9]. The health system includes 10 health regions, 189 health districts, about 1700 health areas, and a community health component in 90 districts, with only 1.07 doctors and nurses per 1000 persons [8, 10]. While the entire country remains at risk for malaria, Cameroon can be divided into multiple epidemiological zones each with varying levels of malaria transmission. For example, the Mapping Malaria Risk in Africa epidemiological stratification divides the country from South to North into three major epidemiological facets: (i) the Equatorial facet, (ii) the Tropical/Sudanian facet that include the North Region, and (iii) the Sahelian facet in the Far North zone [11]. Recent studies have also divided the country into five climate zones: the Sahelian zone (including the Extreme North region), the Soudanian zone (North region), the Sahelo-Guinean zone (Adamaoua region), the Humid Savannah zone (North–West and West regions), and the more southern Forest zone (Central, East, South, Littoral, and South–West regions) [8, 12, 13]. Overall, *Plasmodium falciparum* is estimated to account for > 95% of malaria cases in Cameroon, though *Plasmodium malariae*, *Plasmodium ovale*, and even *Plasmodium vivax* have been detected [13–19].

The economic hub and most populous city of Douala has a smaller representation in the malaria literature than the capital, Yaoundé. Lying within the Littoral Region of the Forest zone, Douala is home to a population of approximately 3 million people [13]. This zone experiences two rainy seasons with annual rainfall between 1500 and 4000 mm and the entomological inoculation rate (EIR) ranges from 0 to 90 infective bites per person per year in Douala [13]. Studies conducted in Douala in 2009–2010 and 2014 estimated the EIR to be 31 and 110, respectively [20, 21]. The Cameroon National Malaria Control Programme reported a country-wide incidence of 103.1 cases per 1000 inhabitants in 2019, with 96.0 cases per 1000 inhabitants in the Littoral Region [22]. The 2018 Demographic and Health Survey (DHS), which focuses on community-based sampling, found 7.8% of children 6–59 months to be RDT-positive for malaria in Douala [23]. Thus, while EIR surveys have suggested high rates of transmission potential in Doula, literature on the prevalence and characteristics of malaria in this populous urban environment are relatively scarce.

Uncomplicated malaria often presents with nonspecific symptoms including malaise, headache, fatigue, abdominal discomfort, and myalgias/arthralgias followed by fever, chills, diaphoresis, anorexia, nausea, and emesis [24]. In infants, malaria may present like any other cause of sepsis with lethargy and poor feeding, and young children may also develop cough or gastrointestinal symptoms [24]. Signs and symptoms of severe malaria include coma, convulsions, impaired consciousness, acute pulmonary edema, renal failure, metabolic acidosis, severe anaemia, or severe hypoglycemia [24], and severe anaemia may be the most common severe manifestation [1]. In settings such as urban Cameroon, anaemia may be caused by several overlapping factors including non-malarial infections, haemoglobinopathies, and poor nutritional status. In a community sample of 6 to 59-month-old children from Douala, the 2018 DHS found a haemoglobin < 11.0 g/dL (anaemia of any severity), 7.0–9.9 g/dL (moderate anaemia), < 8.0 g/dL (regarded as malaria-related anaemia), and < 7.0 g/dL (severe anaemia) in 56.5%, 25.1%, 5.8%, and 1.7% of children, respectively [23]. Individual studies in Douala have examined haemoglobin levels in clinical settings, but comorbidities varied, and different studies used different haemoglobin cutoffs [25–28].

Urban studies looking at rates of malaria and anaemia in those presenting to high level care centers with fever are also less prevalent in the literature. In Cameroon, the few existing studies in Douala over the past 20+ years have been limited by reliance on microscopy for diagnosis, recruitment of a narrow age range, inconsistent haemoglobin thresholds used to define anaemia, and minimal assessment of additional symptomatology. To characterize the burden and presentation of malaria and anaemia in the most populated urban centre in Cameroon, a descriptive survey of symptomatic patients presenting to the Emergency Department (ED) of Douala Military Hospital (DMH) was conducted. This manuscript reports on the prevalence of *P. falciparum* infection and the clinical characteristics of these infections, with a specific emphasis on anaemia.

## Methods

### Study design and study population

A cross-sectional epidemiological and clinical survey of symptomatic patients presenting to the ED of DMH was conducted. The hospital sits in the Douala I district and treats both military and civilian patients. Its ED consists of 2–3 physicians, a triage station staffed by 5–7 nurses, and approximately 18 beds. The study planned to recruit a convenience sample of 450 patients presenting to the ED for whom there was a suspicion of malaria. Recruitment was planned for consecutive, weekday enrollment due to the availability of the laboratory staff and equipment. ED physicians and nurses were asked to refer patients meeting the following eligibility criteria to the study team:

1) Age of 6 months and older.

2) At least one of the following: (a) temperature ≥ 37.5 °C on presentation to the triage nurse station; (b) subjective history of fever in the previous three days; or (c) suspicion of malaria infection by the ED physician.

### Clinical evaluation and point-of-care testing for malaria and anaemia

The recruitment team administered a questionnaire including demographics, travel history, prior medications and traditional remedies used, and symptomology. The team also assessed for clinical signs of anaemia (palmar pallor, conjunctival pallor, and koilonychia). Symptoms and signs were rated on a four-point scale from 0 to 3 with 0 indicating the absence of the symptom/sign, 1 indicating borderline or mild (or once per day in the case of emesis and diarrhoea), 2 indicating moderate (or twice per day for emesis and diarrhoea), and 3 indicating severe symptoms (or ≥ 3 times per day for emesis and diarrhoea). Temperature from the triage station was recorded from the patient’s medical card if available, and an oral temperature (or rectal in infants) was taken by study personnel for all participants.

In the ED, a 2 mL venous blood sample was collected, used to measure haemoglobin level using the HemoCue® 201 + system (HemoCue America, Brea, CA, USA), and tested with a WHO pre-qualified histidine-rich protein 2 (HRP-2)/*Plasmodium* lactate dehydrogenase (pLDH) malaria rapid diagnostic test (RDT) commonly used throughout Cameroon (Standard Q Malaria Pf/Pan Ag RDT, SD Biosensor, Suwon, South Korea) [29].

### Molecular-based speciation from red blood cell pellets

Whole blood samples were centrifuged at 3000 rpm for 5–10 min and then pipetted into plasma and red blood cell (RBC) pellet samples. All samples were stored at -20° C in the DMH lab and later transported on ice to the Malaria Unit of the Centre Pasteur of Cameroon (CPC) in Yaoundé, Cameroon. DNA was extracted from the RBC pellets at the CPC using DNeasy® Blood and Tissue DNA extraction kits (QIAGEN, Germantown, MD, USA) then shipped to the Yale School of Public Health in New Haven, USA on dry ice. Extracted DNA was amplified by nested polymerase chain reaction (PCR) (Additional file 1: Tables S1–2) using GoTaq® Flexi DNA Polymerase kit (Promega Corporation, Madison, WI, USA), according to established protocols with the expected sizes for the species-specific Nest 2 products of 205 bp for *P. falciparum*, 144 bp for *P. malariae*, 120 bp for *P. vivax*, and approximately 800 bp for *P. ovale* [30, 31].

### Data management and statistical analysis

All recruitment data were recorded onto paper standardized case record forms by study staff and then double entered into the database. Participants were grouped into ages < 15 years-old (children) and ≥ 15 years old (adults). Children were further divided into those < 5 years old, 5–9 years old, and 10–14 years old. Detection by PCR constituted the reference standard for the presence of malaria infection. Anaemia was defined by WHO criteria (see Additional file 1: Table S3) based on haemoglobin level (g/dL) assessed with the HemoCue® system [32]. Continuous variables were reported as means ± standard deviation (SD), while categorical variables were reported as number and percent. Means were compared between *P. falciparum*-positive and negative groups using the student t-test, and association between categorical variables and malaria status was assessed using the Pearson Chi-square. Prevalence odds ratios (OR) with 95% confidence intervals (CI) were calculated using binomial logistic regression in which a single predictor variable (e.g. sex) with two categories was entered [33]. Population attributable risk percent (PARP) was defined as (incidence among all participants within the population − incidence among non-exposed)/incidence among all participants within the population. Likelihood ratios (LR) were defined and interpreted according to McGee: the positive LR refers to the LR when the symptom/sign is present and was defined as sensitivity/(1 − specificity), while the negative LR refers to the LR when the symptom/sign is absent and was defined as (1-sensitivity)/specificity [34]. A significant LR was defined as one which changes the probability by ≥ 20%; therefore, a clinically significant positive LR was defined as ≥ 3, while a clinically significant negative LR was defined as ≤ 0.4 (Additional file 1: Table S4) [34]. Statistical significance was defined as 2-tailed p-value < 0.05 for all analyses. Data were analysed in Microsoft Excel and IBM SPSS Version 28 (IBM Corporation, Armonk, NY, USA).

## Results

### Study participant profile

**Fig. 1:**
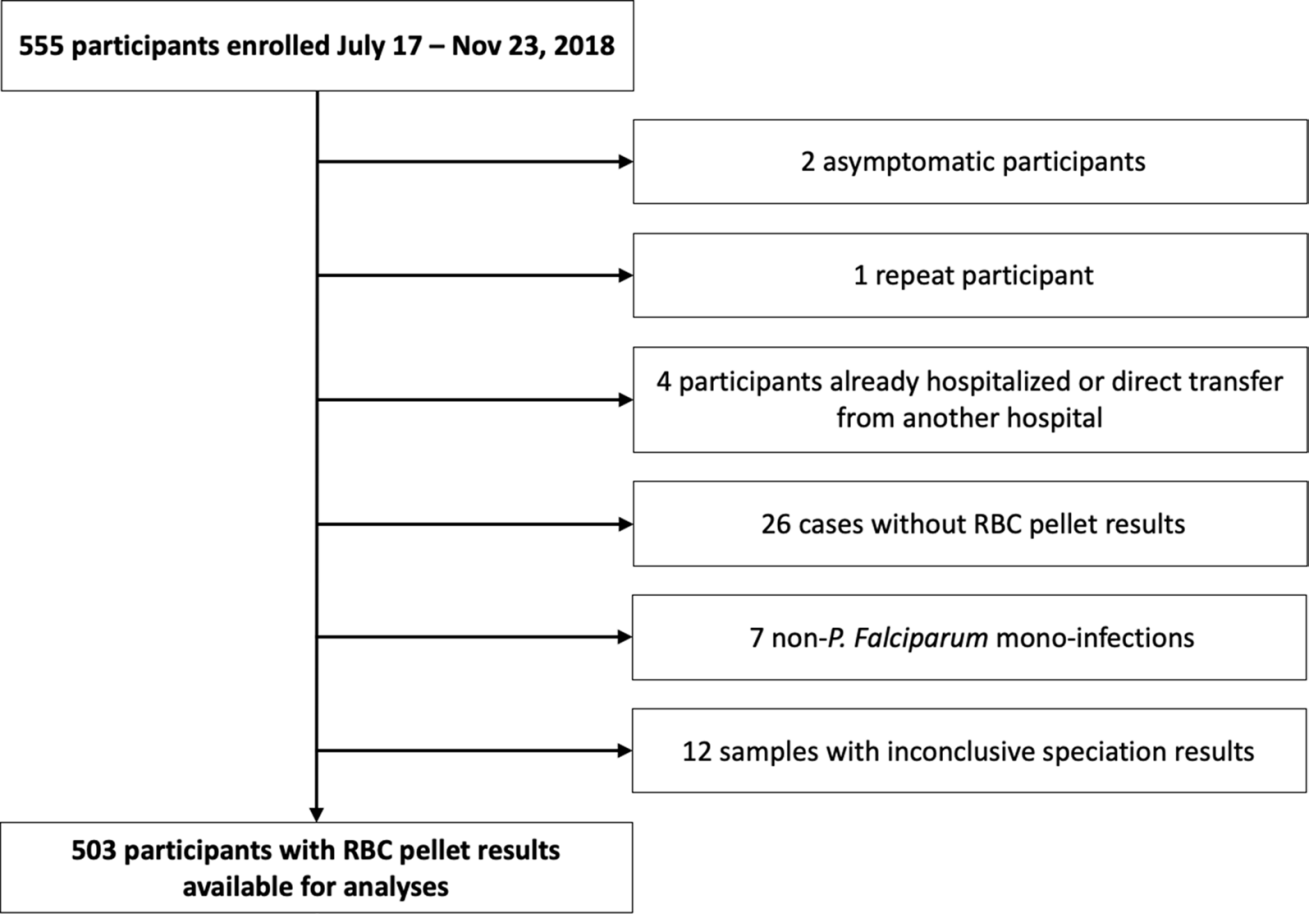
Participant recruitment and inclusion. Diagram describes recruited patients excluded from the analyses for this manuscript. Demographics for the full cohort of recruited patients were similar to the demographics reported here for the 503 participants included in these analyses

Between July 17 and November 23, 2018, a total of 555 participants were enrolled in the study, and 503 results from RBC pellet samples were available for molecular analyses (Fig. 1). Characteristics for these 503 participants are provided in Table 1, and these were similar for the full cohort of recruited participants. Participant sex was balanced, and participant’s mean age was 32 years old (range 8 months to 86 years). There were 7 pregnant women. The cohort represented all districts within Douala: 26.2% (n = 132/503) from Douala I (Bonanjo; site of DMH), 10.5% (n = 53/503) from Douala II (New Bell), 29.4% (n = 148/503) from Douala III (Bonassama), 5.6% (n = 28/503) from Douala IV (Logbaba), 18.9% (n = 95/503) from Douala V (Kotto), and 1.8% (n = 9/503) from the island district of Douala VI (Manoka). Only 3.8% (n = 19/503) of participants were from outside Douala. Over one quarter had traveled outside of Douala during the previous 30 days; the most common destinations included the Central region which includes Yaoundé (9.0%, n = 45/500), the West region (5.8%, n = 29/500) and the greater Littoral region (4.2%, n = 21/500). More participants had already taken an antipyretic (40.3%) than an anti-malarial (17.3%) or a traditional remedy (10.1%).

**Table 1:** Participant characteristics by PCR-determined malaria status

	All n = 503	P. falciparum* positive n = 187	*P. falciparum* negative n = 316	p**
Age	n = 498 32 ± 20 years	n = 186 30 ± 20 years	n = 312 34 ± 20 years	**.038**
Female***	n = 503 257 (51.1)	n = 187 84 (44.9)	n = 316 173 (54.7)	**.033**
Anti-malarial	n = 501 87 (17.4)	n = 186 40 (21.5)	n = 315 47 (14.9)	.060
Antipyretic	n = 501 202 (40.3)	n = 186 86 (46.2)	n = 315 116 (36.8)	**.038**
Traditional remedy	n = 495 50 (10.1)	n = 185 25 (13.5)	n = 310 25 (8.1)	.052
Travel	n = 500 143 (28.6)	n = 185 55 (29.7)	n = 315 88 (27.9)	.668
Fever by history	n = 502 488 (97.2)	n = 186 183 (97.8)	n = 316 306 (96.8)	.505
Fever on presentation	n = 488 322 (66.0)	n = 179 121 (67.6)	n = 309 201 (65.0)	.567
Temperature on presentation	n = 488 37.8 ± 1.0	n = 179 38.1 ± 1.1	n = 309 37.7 ± 0.9	** < .001**

Values reported as mean ± SD for age and number (valid percent) for other variables

Anti-malarial, antipyretic, and traditional remedy refers to participant reported medication use for the current illness episode

Travel refers to participant reported travel within the previous 30 days

Fever by history refers to participant-reported fever within the preceding 72 h. Note that only 2.8% of participants did not have history of fever, and the percentage of participants with history of fever was equivalent between the two groups

Fever on presentation was defined as temperature ≥ 37.5 °C on measurement by either emergency department staff or oral measurement by study personnel

*Includes 9 cases of *P. falciparum*/*P. malariae* and 5 cases of *P. falciparum*/*P. ovale* mixed infections

**Means of continuous variables compared using independent samples t-test and categorical variables compared using Pearson Chi square. Significant p-values < 0.05 bolded

***Includes 7 pregnant women

### Clinical presentation of participants

**Fig. 2:**
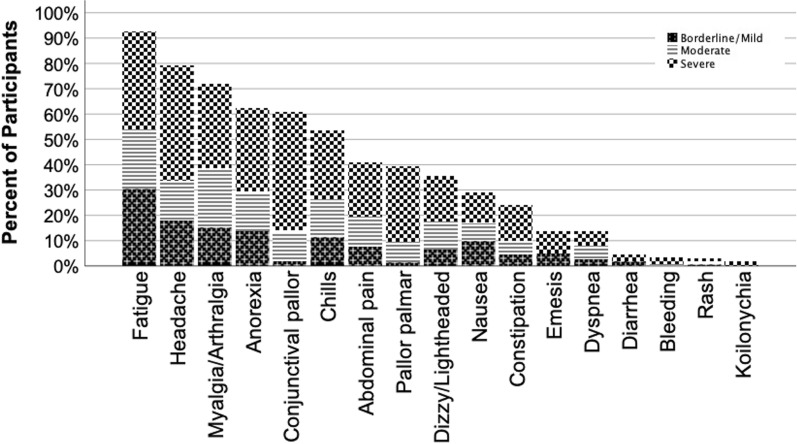
Presenting symptoms and signs. Presenting symptoms and signs of participants. Pattern of shading indicates severity of reported symptom. In addition to the symptoms reported in this figure, 97.2% of participants reported a history of fever in the preceding 72 h

Almost all patients (97.2%, n = 488 / 502) had a history of fever (Table 1), as expected given eligibility criteria. Figure 2 presents the proportion of all participants reporting additional symptoms or with detected signs, as well as the severity of these symptoms and signs. Nonspecific symptoms of fatigue (92.4%, n = 465/503), headache (79.4%, n = 392/494), myalgias/arthralgias (71.8%, n = 359/500), anorexia (62.5%, n = 313/501), and chills (53.5%, n = 268/501) were more frequently reported. Other symptoms were reported by less than half of participants. Among signs of anaemia, conjunctival pallor was observed in 60.7% (n = 304/501), while pallor earning a score of moderate (conjunctival: 11.4%, n = 57/501; palmar: 8.0%, n = 40/501) or severe (conjunctival: 2.0%, n = 10/501; palmar: 1.4%, n = 7/501) was infrequent. Koilonychia was rare and rated as mild/borderline in all cases.

**Fig. 3:**
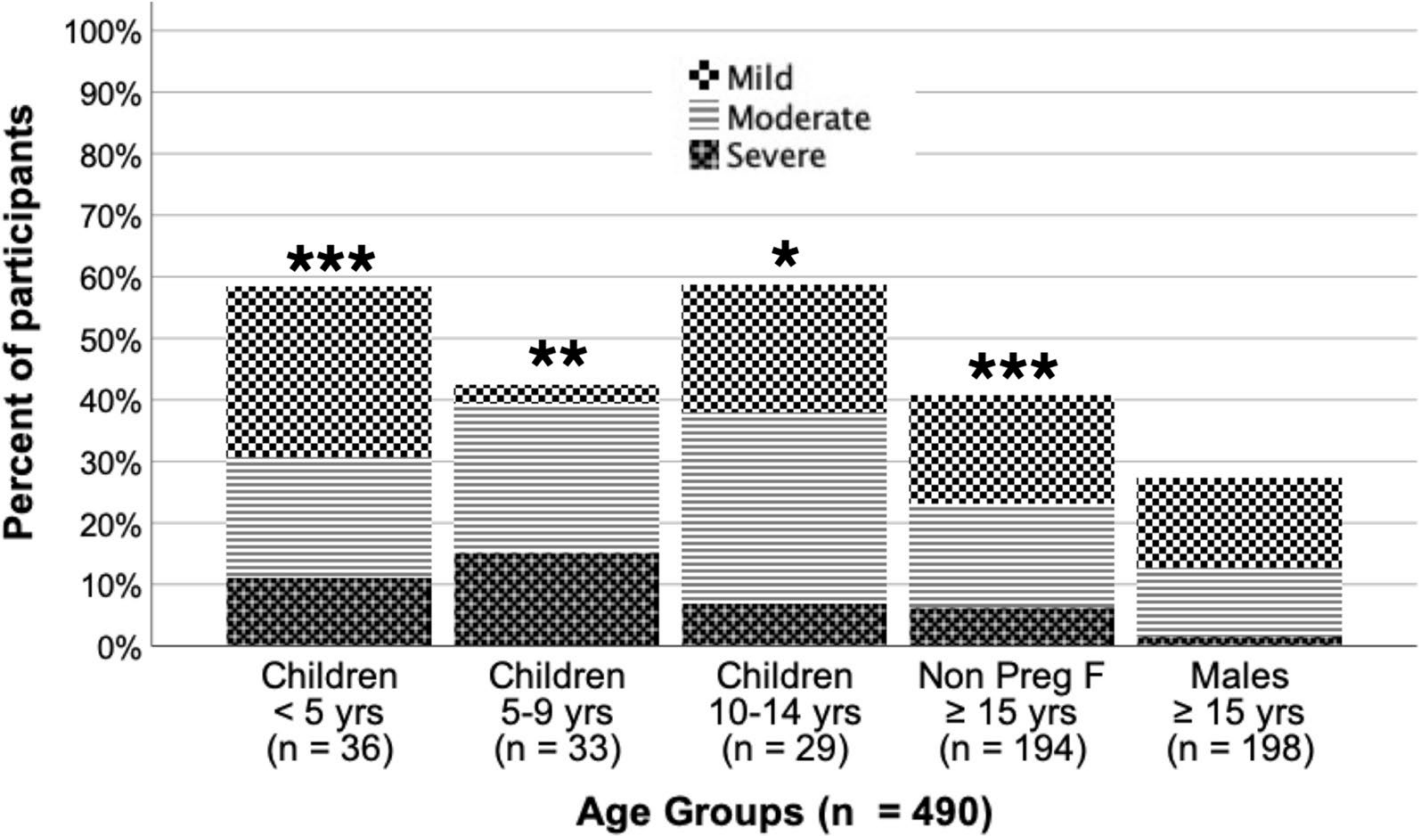
Anaemia by age groups. Percent of participants with anaemia by age group. For participants 15 years old, the group is split between males and females with pregnant women excluded. Shading pattern indicates anaemia severity. See text for complete details. N Preg F: non pregnant females. *Indicates anaemia (any severity) was significant higher compared to males ≥ 15 years old. **Indicates severe anaemia was significantly higher compared to males ≥ 15 years old. *** Indicates both anaemia (any severity) and severe anaemia were higher compared to males ≥ 15 years old

Among all participants, the prevalence of anaemia was 38.0% (n = 189/497), including 16.3% (n = 81/497) with moderate and 5.2% (n = 26/497) with severe anemia. Clinical findings associated with anaemia can be found in Additional file 1: Table S5. Four participants under the age of 12 had a haemoglobin < 5 g/dL, and 8 participants ≥ 12 years old had a haemoglobin < 7 g/dL, which are part of the criteria for severe malaria based on the presence of low haemoglobin. The prevalence of anaemia (any severity) was significantly higher (OR: 2.20, 95% CI 1.41–3.45) among children < 15 years (53.1%, n = 52/98) compared to adults ≥ 15 years old (34%, n = 133/392). The prevalence of severe anaemia was also significantly higher (OR: 3.18, 95% CI 1.41–7.16) among children (11.2%, n = 11/98) compared to adults (3.8%, n = 15/392). Considering different age groups, anaemia (any severity) was significantly higher among children < 5 years old (OR: 3.73, 95% CI 1.79–7.77), children 10–14 years (OR: 3.78, 95% CI 1.69–8.43), and nonpregnant women ≥ 15 years old (OR: 1.83, 95% CI 1.20–2.80) compared to men ≥ 15 years old (Fig. 3). Furthermore, severe anaemia was significantly higher among children < 5 years old (OR: 8.13, 95% CI 1.74–38.01), children 5–9 years (OR: 11.61, 95% CI 2.63–51.25), and nonpregnant women ≥ 15 years old (OR: 4.29, 95% CI 1.19–15.43) compared to men ≥ 15 years old (Fig. 3).

### Diagnosis of *P. falciparum* infection

One quarter (25.2%, n = 126/500) of participants were positive by RDT, with 12.2% (n = 61/500) positive for HRP-2 only (*P. falciparum* infection only), 12.6% (n = 63/500) positive for both HRP-2 and pLDH (*P. falciparum* mono- or mixed infection), and only 0.4% (n = 2/500) positive for pLDH alone (either mono- or mixed non-*P. falciparum* species infection).

**Fig. 4:**
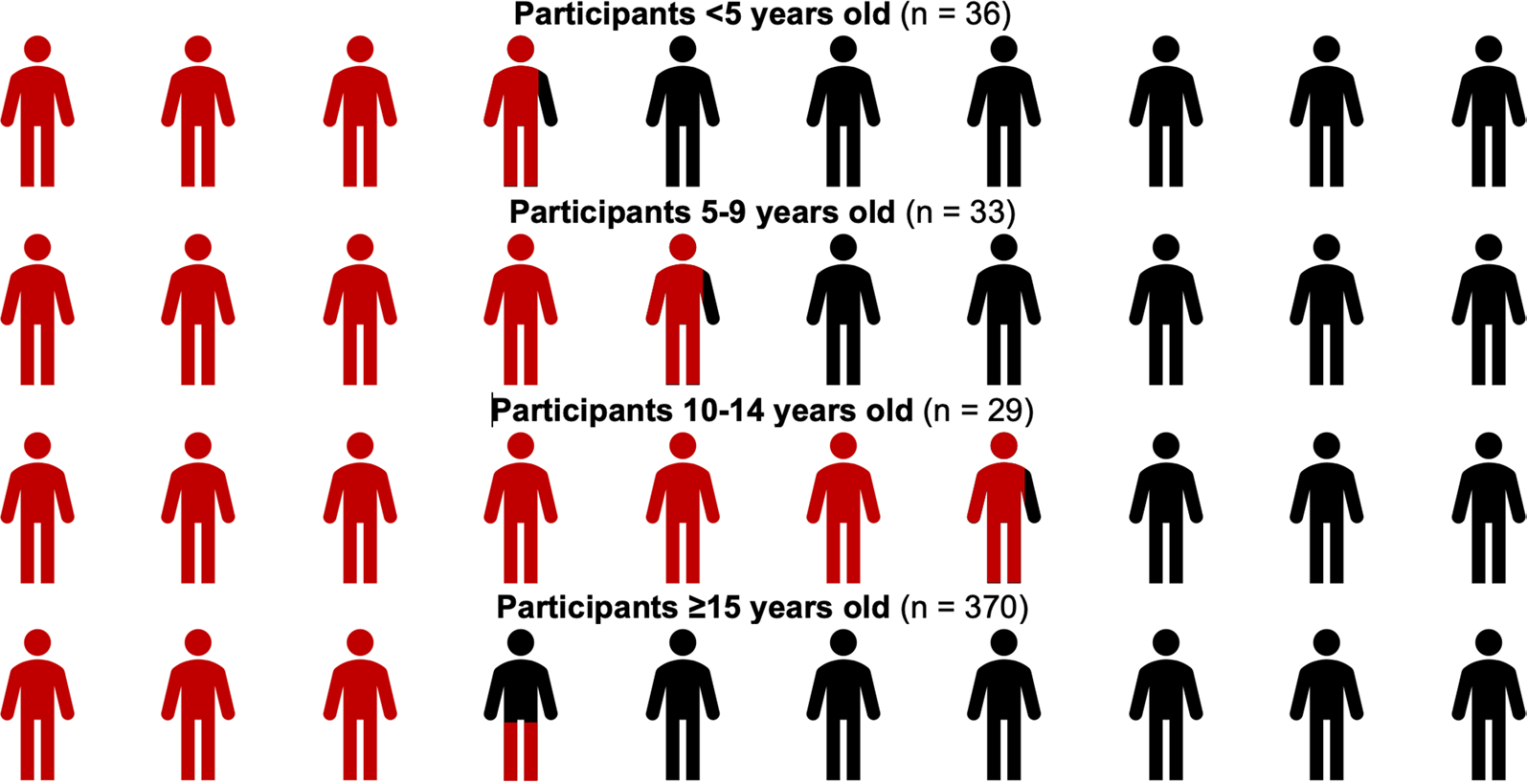
*Plasmodium falciparum* infection by age group. Each stick-figure represents 10% of participants in that age group, and red indicates *P. falciparum* was detected by PCR. The prevalence of *P. falciparum* was significantly higher among participants aged 10–14 compared to participants < 5 years old and compared to participants ≥ 15 years old. See text for complete details

Malaria rates were higher by nested PCR; among all participants, 37.2% (n = 187/503) were positive for *P. falciparum*, including 34.4% (n = 173/503) *P. falciparum* mono-infection, 1.8% (n = 9/503) *P. falciparum*/*P. malariae* mixed infection, and 1.0% (n = 5/503) *P. falciparum*/*P. ovale* mixed infection. *P. falciparum* infection status varied by age group (Fig. 4), with *P. falciparum* detected by PCR in 38.9% (n = 14/36) of < 5 year-olds, 48.5% (n = 16/33) of 5–9 year-olds, 69.0% (n = 20/29) of 10–14 year-olds, and 34.0% (n = 136/400) of ≥ 15 years old. There was a significant association between age group and either positive RDT (χ^2^(3) = 31.31, p < 0.001) or detection of *P. falciparum* by PCR (χ^2^(3) = 16.09, p < 0.001) with the highest standardized residuals for the 10–14-year-old age group. The prevalence of *P. falciparum* as diagnosed by PCR was significantly higher among participants aged 10–14 compared to participants < 5 years old (OR: 3.49, 95% CI 1.24–9.82) and ≥ 15 years old (OR: 4.31, 95% CI 1.91–9.73).

#### Comparison to DHS data

Regarding measurement of haemoglobin level among children 6–59 months old, haemoglobin was < 11.0 g/dL in 58.3% (compared to 56.5% in the DHS), between 7.0–9.9 g/dL in 19.4% (compared to 25.1%), < 8.0 g/dL in 13.9% (compared to 5.8%), and < 7.0 g/dL in 11.1% (compared to 1.7%). Regarding prevalence of malaria infection as diagnosed by RDT, 25.0% of participants 6–59 months were RDT positive, compared to 7.8% in the DHS.

### Characteristics of *P. falciparum* PCR-positive participants

PCR results were used as the reference standard for malaria diagnosis. Compared to PCR-negative participants, *P. falciparum* PCR-positive participants were younger (t = 2.08, p = 0.038) and more likely to have reported already trying an antipyretic (χ^2^(1) = 4.31, p = 0.038) (Table 1). Presenting temperature was also higher for *P. falciparum-*positive versus negative participants (t = − 3.6, p < 0.001). Among epidemiological factors, clinically significant positive LRs for *P. falciparum* infection by PCR were associated with age 10–14 years (positive LR: 3.73), living in the island district of Douala VI (positive LR: 3.41), and travel to any of three northern regions (positive LR: 5.11). Clinically, high fever > 40 °C at the time of presentation (positive LR: 4.83) and frequent emesis ≥ 2 times per day (19.85) were associated with clinically significant positive LRs. Several other gastrointestinal symptoms weakly increased the probability of *P. falciparum* infection with positive LR >2.0 (moderate to severe abdominal pain, nausea, or diarrhoea). Considering PCR as the gold standard, RDTs demonstrated high positive and negative predictive value in this urban setting. A positive RDT (any band) increased the probability of malaria dramatically (positive LR: 28.70), and an HRP-2/pLDH positive result had a positive predictive value close to 1.0 (positive predictive value: 0.997, positive LR > 100). The absence of a positive RDT (i.e., a negative RDT) was associated with a clinically significant negative LR (0.37). Table 2 presents likelihood ratios separately for children and adults. Notably, moderate to severe anaemia significantly increased the probability of *P. falciparum* infection among children (positive LR: 3.24–4.32), but not adults (positive LR: 0.70–0.91).

**Table 2:** Diagnostic characteristics of clinical findings for PCR-confirmed *P. falciparum infection*

	Children < 15 years old (n = 98)					Adults ≥ 15 years old (n = 400)				
	n	Sensitivity	Specificity	Positive LR	Negative LR	n	Sensitivity	Specificity	Positive LR	Negative LR
*Epidemiological history*										
Sex	98									
Female		.560	.479	1.08	.92	400	.404	.443	.73	1.34
District of residence	94					385				
Douala I		.213	.574	.50	1.37		.168	.685	.53	1.21
Douala II		.191	.915	2.25	.88		.130	.913	.91	.95
Douala III		.383	.766	1.64	.81		.298	.697	.98	1.01
Douala IV		.021	.957	.50	1.02		.038	.925	.51	1.04
Douala V		.128	.809	.68	1.08		.252	.815	1.36	.92
Douala VI		.043	1.000	1.00	.96		.031	.988	2.59	.98
Outside Douala		.021	.979		1.00		.084	.976	**3.56**	.94
Travel	98					397				
Anywhere		.160	.792	.77	1.06		.343	.707	1.17	.93
Northern Regions		.040	1.000	***	.96		.030	.992	**3.93**	.98
Medication use						398				
Antipyretic	98	.580	.667	1.74	.63		.415	.620	1.09	.94
Antimalarial	98	.280	.854	1.92	.84		.193	.852	1.30	.95
Traditional remedy	97	.061	.917	.74	1.02		.156	.919	1.91	.92
*Clinical history*										
Fever at presentation	90					393				
≥ 37.5 °C		.787	.326	1.17	.65		.641	.355	.99	1.01
≥ 40.0 °C		.149	.953	**3.20**	.89		.053	.989	**4.67**	.96
Fatigue	98					400				
Any severity		.940	.125	1.07	.48		.926	.072	1.00	1.02
Moderate-Severe		.740	.479	1.42	.54		.610	.538	1.32	.72
Headache	90					399				
Any severity		.812	.217	1.04	.86		.837	.186	1.03	.88
Moderate-Severe		.489	.867	**3.67**	.59		.452	.712	1.57	.77
Myalgias/Arthralgias	96					399				
Any severity		.540	.652	1.55	.71		.800	.231	1.04	.87
Moderate-Severe		.240	.891	2.21	.85		.526	.610	1.35	.78
Anorexia	98					398				
Any severity		.840	.417	1.44	.38		.662	.424	1.15	.80
Moderate-Severe		.600	.688	1.92	.58		.360	.802	1.82	.80
Chills	98					398				
Any severity		.660	.525	1.76	.54		.596	.496	1.18	.82
Moderate-Severe		.400	.833	2.40	.72		.375	.798	1.86	.78
Abdominal pain	95					397				
Any severity		.633	.478	1.21	.77		.511	.706	1.74	.69
Moderate-Severe		.408	.848	2.68	.70		.259	.878	2.12	.84
Dizzy/Lightheaded	94					399				
Any severity		.479	.783	2.20	.67		.422	.674	1.30	.86
Moderate-Severe		.354	.870	2.72	.74		.178	.848	1.17	.97
Nausea	94					396				
Any severity		.490	.733	1.84	.70		.368	.781	1.68	.81
Moderate-Severe		.449	.911	**5.05**	.60		.206	.889	1.86	.89
Constipation	96					398				
Any severity		.306	.830	1.80	.84		.200	.741	.774	1.08
Moderate-Severe		.143	.894	1.34	.96		.104	.909	1.14	.99
Emesis	88									
Any frequency		.386	.818	2.13	.75	381	.161	.922	2.07	.91
≥ 2 times/day		.250	.977	**11.00**	.77		.089	.996	**22.80**	.91
Dyspnea	95					398				
Any severity		.125	.936	1.96	.93		.215	.886	1.88	.89
Moderate-Severe		.104	.957	2.45	.94		.111	.935	1.70	.95
Diarrhoea	96					385				
Any frequency		.104	.958	2.50	.93		.055	.969	1.76	.98
≥ 2 times/day		.042	1.000	*** (2)	.96		.023	.098	1.51	.99
Bleeding/Haemorrhage	97					393				
Any severity		.061	.958	1.47	.98		.038	.98	1.61	.99
Moderate-Severe		.020	1.000	*** (1)	.98		.000	.992	1.96	.99
Cutaneous eruptions	97					399				
Any severity		.000	.915	.000	1.09		.044	.981	2.35	.97
Moderate-Severe		.000	.957	.000	1.04		.015	.992	1.96	.99
*Point of care testing*										
RDT	98					397				
Any positive		.800	.958	**19.20**	**0.21**		.585	.981	**30.66**	.42
HRP-2 positive		.360	.979	**17.28**	0.65		.274	.981	**14.36**	.74
Both HRP-2/LDH positive		.440	.979	**21.12**	0.57		.296	1.00	*** (40)	.70
Anaemia	98					399				
Any severity		.700	.646	1.98	.56		.338	.654	.98	1.01
Moderate-Severe		.540	.833	**3.24**	.55		.169	.814	.91	1.02
Severe		.180	.958	**4.32**	.86		.029	.958	.70	1.01

LR: likelihood ratio. Clinically significant likelihood ratios bolded

^***^Indicated LR could not be calculated as all the patients (number listed in parentheses) with the symptom/sign were found to be *P. falciparum* positive

Northern regions include Adamaoua, North, and Far-North

**Fig. 5:**
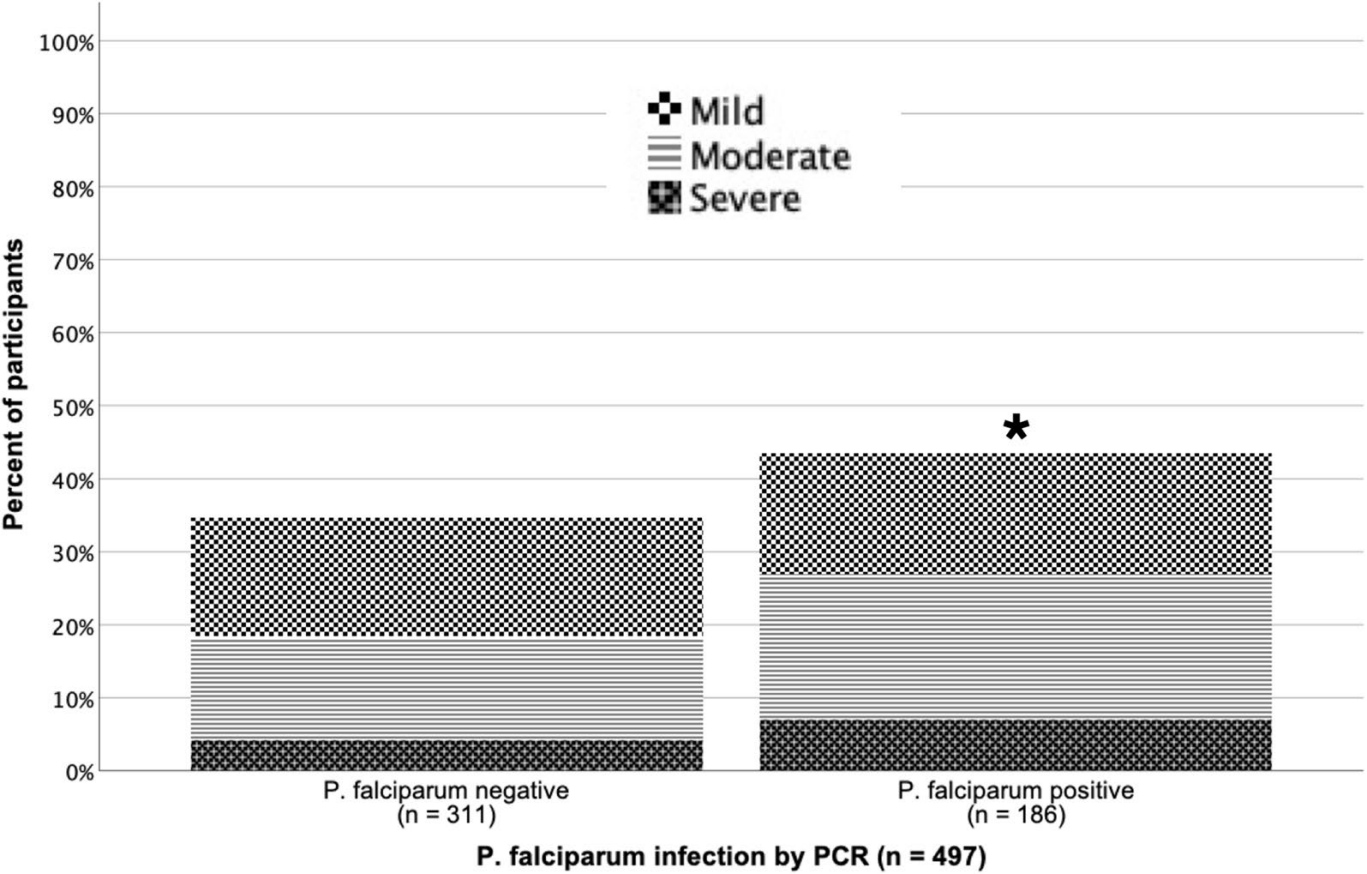
Anaemia by *P. falciparum* infection status. Percent of participants with anaemia by *P. falciparum* infection status. Pattern of shading indicates anaemia severity. See text for complete details. *Indicates moderate-severe anaemia was significantly higher among *P. falciparum* positive participants

Among all participants, 8.7% (the PARP) of anaemia (any severity) was associated with *P. falciparum* infection, but this varied dramatically by age as discussed below. The prevalence of moderate to severe anaemia was significantly higher (OR: 1.64, 95% CI 1.06–2.53) among *P. falciparum*-positive (26.9%, n = 50/186) versus *P. falciparum*-negative participants (18.3%, n = 57/311), and the association between overall rates of anaemia and *P. falciparum* infection trended toward statistical significance (OR: 1.45, 95% CI 1.00–2.10) (Fig. 5b). Among children < 15 years old, 33.3% of anaemia was associated with *P. falciparum* infection, and the prevalence of anaemia (of any severity) was significantly higher (OR: 4.26, 95% CI 1.83–9.92) among participants with *P. falciparum* (70.0%, n = 35/50) compared to those negative for *P. falciparum* (35.4%, n = 17/48). For those ≥ 15 years old, anaemia was not associated with malaria (PARP -0.8%) and anaemia rates did not differ statistically by *P. falciparum* infection status (OR: 0.97, 95% CI 0.62–1.50). The percent of anaemia associated with *P. falciparum* infection varied by age, as the PARP was 14.3% among children < 5 years old, 30.7% among children 5–9-year-olds, and 81.0% among 10–14-year-olds.

## Discussion

An 18-week cross-sectional study of symptomatic patients across the age spectrum presenting to an urban hospital in the most populous city in Cameroon was conducted and found a high incidence of both *P. falciparum* infection and anaemia. As DMH is a fee-for-service hospital in a major metropolitan area, a lower prevalence of malaria infection may have been expected, as such centres receive febrile patients with a variety of aetiologies; however, over one third of individuals presenting with fever, history of fever, or other suspicion for malaria were PCR-positive for *P. falciparum*. This study echoes findings from an urban hospital in Sierra Leone during the same year [35]. In that study, febrile patients presenting to the hospital were found to have 50% positivity for malaria, overwhelmingly *P. falciparum*, using a highly sensitive multiplex real-time PCR assay. Recent research shows that new vectors may emerge in large, urban environments [3, 36] and that malaria transmission may have actually increased within cities across sub-Saharan Africa in the new millennium [5]. Within the context of these broader trends, these findings emphasize the need for proper surveillance and recognition that incidence of *P. falciparum* infection may remain high even within the largest population centres traditionally felt to be at lower risk.

Several epidemiologic and clinical characteristics were associated with a higher probability of *P. falciparum* infection among these participants. Residence on the island district of Manoka (Douala VI) or travel to any of the three northern regions (Adamaoua, North, or Far-North) increased the probability of *P. falciparum* detection. Travel as an important risk factor among an urban population has been previously described in Malawi [6]. Children 10–14 years of age were more likely than children < 5 years old to be *P. falciparum* positive, which may reflect broader trends in higher prevalence among older children across sub-Saharan Africa [7, 37]. Possible explanations for these trends in similar settings include the targeting of malaria control measures (e.g. bed nets, seasonal malaria chemoprevention) and the distribution of antimalarials at no charge to children under 5 years of age in many countries. Among all participants, high fever > 40 °C at presentation and frequent emesis increased the probability of *P. falciparum* infection. Among children, more severe symptoms generally (headache, emesis, diarrhaea) increased the probability of *P. falciparum*. Importantly, while more severe degrees of anaemia increased the probability of *P. falciparum* infection among children < 15 years old, this was not seen among participants ≥ 15 years old.

Applying these results to children < 15 years old, the prevalence (pre-test probability) of *P. falciparum* infection was 51%, and a child presenting with fever > 40 °C and severe anaemia would have over 95% probability of *P. falciparum* infection. Among adults ≥ 15 years old, the prevalence (pre-test probability) of *P. falciparum* infection was lower at 34%. However, an individual in this age group presenting with frequent emesis and recent travel to the North would have a near 100% probability of *P. falciparum* infection. While not a replacement for malaria diagnostic tests, a better understanding of characteristic epidemiological and clinical trends can help health care providers risk stratify patients to streamline testing and treatment. Criteria and calculators to stratify risk for other infections are already employed in urgent care and emergency settings, for example the Centor score for *Streptococcal* pharyngitis [38] or the Alvarado score for appendicitis [39]. Creating site specific criteria for individual health care centres in high-transmission zones could prove a powerful tool in the clinical management of malaria.

In this cohort, over 17% had already taken an antimalarial, and among these, fewer than 50% were found to have *P. falciparum* infection by PCR testing. Self-medication prior to presentation to the hospital is quite common. Private pharmacies are proliferating in urban centres, where most drugs are available without a need for clinical consultation. A recent survey of customers at pharmacies in Douala IV found 47% of customers buying antibiotics did so without a prescription [40]. Determining which medication was taken can be challenging, and home treatment can clearly affect both presenting symptomology and the results of diagnostic testing. Self-medication, therefore, complicates the diagnostic picture for clinicians, and it is critical for clinicians to inquire about prior medication usage when evaluating patients.

Almost 40% of participants had anemia, with > 5% meeting criteria for severe anemia. While the burden of anaemia was not confined to younger age groups, the prevalence of anaemia was higher among children and women compared to adult men. In comparison to the community sample of children < 5 years old in the 2018 DHS, there was a higher percentage of more severe anaemia [23]. Such a difference may reflect the different populations from which participants were drawn, as the current study targeted symptomatic patients who were presenting to an ED and much more likely to have clinical illness that might result in anaemia. In comparison to other clinical samples, one older study in Douala assessed anaemia among microscopy-positive children < 15 years old and found 28.7% had a haemoglobin < 8 g/dL [25]. Other studies have investigated haemoglobin status in malaria patients with and without the comorbidities of sickle cell disease and HIV in Douala. One study of patients with malaria and HIV found the overall prevalence of haemoglobin < 11 g/dL was 28.5%, and the prevalence among malaria mono-infected individuals and among malaria/HIV co-infected individuals was 20.4% and 43.3%, respectively [27]. In a different study, 6.7% of the non-sickle cell malaria patients had a haemoglobin < 5 g/dL [28], while in yet another study, no patients with malaria infection without HIV had a haemoglobin < 7 g/dL [27]. While direct comparison across clinical settings is difficult, the results of the current study and others highlight the high level of burden of anaemia among inhabitants of Douala, particularly children.

This study further demonstrates that a considerable risk for anaemia among symptomatic children is associated with malaria infection. Considering 10–14-year-olds for example, this group had a higher prevalence of *P. falciparum* infection than young children or adults, and over 80% of anaemia was associated with malaria infection. The role of malaria in the burden of anaemia in Cameroon may extend even to asymptomatic malaria infection as a recent longitudinal study across multiple villages in Cameroon’s Central Region found asymptomatic malaria infection (diagnosed by PCR) was associated with 69.2% of anaemia in the study population [41]. Despite this association, the relationship between anaemia and malaria in children remains complex. Iron deficiency anaemia may be associated with decreased risk of malaria infection and clinical illness in children, while universal iron and folate supplementation in children may lead to worse outcomes [42, 43]. Certain anti-malarials can also result in anemia; for example primaquine can result in acute haemolytic anaemia in patients with glucose-6-phosphate dehydrogenase deficiency [44].

These findings have implications for clinical practice. First, effectively detecting and treating malaria infection may reduce the overall burden of anaemia among children. Indeed, chemoprophylaxis against malaria was recently found to reduce death and readmissions among children admitted for severe anaemia [45]. Second, the risk factors for anaemia in the setting of malaria infection need to be better understood. In Cameroon, some of these factors may include parasitemia, duration of clinical illness, presence of stunting, and preexisting iron deficiency [46]. Third, follow-up haemoglobin/haematocrit measurements may be warranted following recovery from malaria to determine the need for additional workup or intervention. In Cameroon, longitudinal follow up of children ≤ 14 years old in whom both *P. falciparum* and anaemia were detected has shown that over 50% may remain with low haemoglobin even weeks after treatment for malaria [46]. While iron supplementation may not be recommended during acute infections during the high transmission season, iron repletion may be beneficial during the low transmission season [47].

There are important limitations to this study. First, parasitaemia levels by microscopy or quantitative PCR were not available, and the patient’s final diagnosis was not known. Thus, some percentage of *P. falciparum* infections were either incidental findings or one or multiple comorbid infections responsible for the patient’s febrile illness. This study can therefore only comment on prevalence of *P. falciparum* infection rather than the clinical entity of *P. falciparum* malaria, including severe malaria. Second, recruitment took place at a single urban site over less than half the year, specifically during and after the summer rainy season when malaria transmission would be expected to be highest, and only during weekdays when laboratory resources were available. The DMH itself is a private, fee-for-service secondary care facility located proximal to major government facilities, and it serves as the regional medical centre for the Cameroonian Army, therefore serving a unique client base. Thus, participants in this study likely do not reflect the population of Douala as a whole. Third, the study was not powered to test specific hypotheses, and the small number of participants in younger age groups greatly limited the power of the study to detect differences among these age groups. Fourth, additional laboratory data to classify the type of anaemia was not obtained, nor were laboratory tests for haemoglobinopathies or helminth infections performed. As mentioned above, there are a myriad of possible etiologies for anaemia among these participants. Thus, this study cannot identify the etiology of anaemia among the participants and can only comment on possible association, rather than causation, between malaria and anemia. Finally, the assessment of presenting symptoms was subjective.

## Conclusions

The current study describes clinical characteristics of *P. falciparum* infection in Douala, Cameroon. A high burden of both *P. falciparum* infection and anaemia was found in this populous urban setting. Older children tended to be at higher risk for *P. falciparum* infection, and several clinical and epidemiologic characteristics significantly increased the likelihood of malaria infection. Anaemia was higher among children and woman compared to adult men, and the presence of anaemia was associated with *P. falciparum* infection only among children. With recent slowing or even reversal of gains in malaria control efforts, additional epidemiologic studies in urban centres as well as across the full age spectrum in sub-Saharan Africa are recommended, so that appropriate interventions can be developed and deployed for these unique settings and demographics.

## Supplementary Information

**Additional file 1:** **Table S1.** PCR Mastermix reagents and concentrations for PCR speciation assays.** Table S2.** PCR amplification parameters for PCR speciation assays. **Table S3. **WHO haemoglobin classification of anaemia.** Table S4. **Interpreting likelihood ratios.** Table S5.** Diagnostic characteristics of history and physical findings for anaemia.

## Data Availability

The datasets generated and analysed during the current study are available from the corresponding author on reasonable request.

## Abbreviations

CI: Confidence interval
CPC: Centre Pasteur of Cameroon
DHS: Demographic and Health Survey
DMH: Douala Military Hospital
ED: Emergency Department
EIR: Entomological inoculation rate
HRP-2: Histidine rich-protein 2
LR: Likelihood ratio
OR: Odds ratio
PARP: Population attributable risk percent
PCR: Polymerase chain reaction
RBC: Red blood cell
SD: Standard deviation
RDT: Rapid diagnostic test
WHO: World Health Organization
