# Supplementary material for: Clinical characteristics of Plasmodium falciparum infection among symptomatic patients presenting to a major urban military hospital in Cameroon
Source: Malar J. 2022 Oct 22;21:298. doi: 10.1186/s12936-022-04315-2 (PMC9588226; doi:10.1186/s12936-022-04315-2)
Supplement: Supplementary file 1 — Additional file 1: Table S1. PCR Mastermix reagents and concentrations for PCR speciation assays. Table S2. PCR amplification parameters for PCR speciation assays. Table S3. WHO haemoglobin classification of anaemia. Table S4. Interpreting likelihood ratios. Table S5. Diagnostic characteristics of history and physical findings for anaemia. [file 12936_2022_4315_MOESM1_ESM.docx]

**Supplemental Materials**

**Supplemental Table 1: PCR Mastermix reagents and concentrations for PCR speciation assays**

| **Reagent** | **Concentration** | **Vol/Rxn**  **(**µL) |
| --- | --- | --- |
| *H2O* | NA | 6.40 |
| *5x GoTaq Buffer* | 1x | 2.50 |
| *25 mM MgCl* | 1.5 mM | 0.75 |
| *10 mM dNTP* | 0.2 mM | 0.25 |
| *10 µM forward primer* | 0.2 µM | 0.25 |
| *10 µM reverse primer* | 0.2 µM | 0.25 |
| *GoTaq polymerase* | 1U/rxn | 0.10 |
| *Template DNA* | ≤ 1 µg/rxn | 2.00 (total) |
| Vol/Rxn: Volume/Reaction  dH2O: nuclease free water  mM: millimolar  µM: micromolar | | |

**Supplemental Table 2: PCR amplification parameters for PCR speciation assays**

| **Cycle** | **Temperature** | **Time** | **Cycles**  **Nest 1** | **Cycles**  **Nest 2** |
| --- | --- | --- | --- | --- |
| *Initial denaturing* | 94 C | 1:00 | 1 | 1 |
| *Denaturing* | 94 C | 1:00 | 35 | 30 |
| *Annealing* | 58 C | 2:00 | 35 | 30 |
| *Extension* | 72 C | 5:00 | 35 | 30 |
| *Final extension* | 72 C | 5:00 | 1 | 1 |
| *Infinite hold* | 4 C | ∞ | - | - |

**Supplemental Table 3: WHO haemoglobin classification of anaemia**

|  | **No**  **anaemia** | **Mild**  **anaemia** | **Moderate**  **anaemia** | **Severe**  **anaemia** |
| --- | --- | --- | --- | --- |
| Children 6-59 months | ≥ 11.0 | 10.0–10.9 | 7.0–9.9 | < 7.0 |
| Children 5-11 years | ≥ 11.5 | 11.0–11.4 | 8.0–10.9 | < 8.0 |
| Children 12-14 years | ≥ 12.0 | 11.0–11.9 | 8.0–10.9 | < 8.0 |
| Women ≥ 15+ years | ≥ 12.0 | 11.0–11.9 | 8.0–10.9 | < 8.0 |
| Pregnant women | ≥ 11.0 | 10.0–10.9 | 7.0–9.9 | < 7.0 |
| Men ≥ 15 years | ≥ 13.0 | 11.0–12.9 | 8.0–10.9 | < 8.0 |
| Haemoglobin reported in g/dL. Adapted from: Haemoglobin concentrations for the diagnosis of anaemia and assessment of severity. Geneva: World Health Organization; 2011; 3 (Table 1). | | | | |

**Supplemental Table 4: Interpreting likelihood ratios**

| **Positive LR** | **Change in probability** | **Negative LR** |
| --- | --- | --- |
| 1 | No change | 1 |
| 2 | +/- 15% | 0.5 |
| **3** | **+/- 20%** | **0.4** |
| **4** | **+/- 25%** | **0.3** |
| **5** | **+/- 30%** | **0.2** |
| **6** | **+/- 35%** | **-** |
| **7** | **+/- 35–39%** | **-** |
| **8** | **+/- 40%** | **0.1** |
| **9** | **+/- 41–44%** | **-** |
| **10** | **+/- 45%** | **-** |
| LR: likelihood ratio. Clinically significant LRs bolded.  Adapted from McGee S. Evidence-Based Physical Diagnosis 4th Edition. Philadelphia, PA: Elsevier; 2018; 14 (Table 2). | | |

**Supplemental Table 5: Diagnostic characteristics of history and physical findings for anaemia**

|  | Sensitivity | Specificity | Positive LR | Negative LR |
| --- | --- | --- | --- | --- |
| Fatigue (n = 497)  *Any severity*  *Moderate-Severe* | .931  .582 | .081  .494 | 1.01  1.15 | .66  .85 |
| Conjunctival pallor (n = 495)  *Any severity*  *Moderate-Severe* | .739  .293 | .479  .964 | 1.42  **8.17** | .54  .73 |
| Palmar pallor (n = 495)  *Any severity*  *Moderate-Severe* | .537  .213 | .700  .977 | 1.79  **9.33** | .66  .81 |
| Dizzy/Lightheaded (n = 492)  *Any severity*  *Moderate-Severe* | .384  .211 | .661  .847 | 1.13  1.38 | .93  .93 |
| Dyspnea (n = 492)  *Any severity*  *Moderate-Severe* | .163  .103 | .880  .935 | 1.36  1.59 | .95  .96 |
| Koilonychia (n = 493)  *Any severity*  *Moderate-Severe* | .037  - | .993  - | **5.73**  - | .97  - |
| Anaemia defined by WHO criteria (see Supplemental Table 3) based on haemoglobin level assessed with the HemoCue® system.  LR: likelihood ratio. Clinically significant likelihood ratios bolded. | | | | |
